# Feasibility testing of a community dialogue approach for promoting the uptake of family planning and contraceptive services in Zambia

**DOI:** 10.1186/s12913-020-05589-5

**Published:** 2020-08-08

**Authors:** Margarate Nzala Munakampe, Theresa Nkole, Adam Silumbwe, Joseph Mumba Zulu, Joanna Paula Cordero, Petrus S. Steyn

**Affiliations:** 1 Department of Health Policy and Management, University of Zambia, School of Public Health, Lusaka, Zambia; 2 Strategic Centre for Health Systems Metrics & Evaluations (SCHEME), School of Public Health, Department of Epidemiology & Biostatistics, University of Zambia, Lusaka, Zambia; 3 Levy Mwanawasa Medical University (LMMU), Lusaka, Zambia; 4 UNDP-UNFPA-UNICEF-WHO-World Bank Special Programme of Research, Development and Research Training in Human Reproduction (HRP), Department of Sexual and Reproductive Health and Research, World Health Organization (WHO), Geneva, Switzerland

**Keywords:** Family planning and contraception, Community participation, Contraception, Community dialogue, Zambia

## Abstract

**Background:**

Community dialogues have been used in participatory approaches in various health prevention and awareness programs, including family planning interventions, to increase understanding and alignment of particular issues from different peoples’ perspectives. The main objective of this paper is to document the feasibility of a community dialogue approach, which aimed to promote dialogue between healthcare providers and community members. The feasibility testing was part of formative-phase research needed to design an intervention, with the ultimate goal of increasing the uptake of family planning and contraception. The community dialogue intervention generated discussions on key approaches to improve family planning and contraception provision and uptake.

**Methods:**

Key stages of the community dialogue were undertaken, with representation from healthcare providers and community members. Participants included frontline and managerial health care providers, community health workers, family planning and contraception users, the youth, other stakeholders from the education sector, and civil society. How the dialogue was implemented (operational feasibility) as well as the cultural feasibility of the community dialogue content was evaluated through participant observations during the dialogue, using a standardised feasibility testing tick-list, and through focus group discussions with the stakeholders who participated in the community dialogue.

**Results:**

Overall, 21 of the 30 invited participants attended the meeting- 70% attendance. The approach facilitated discussions on how quality care could be achieved in family planning and contraception provision, guided by the ground rules that were agreed upon by the different stakeholders. A need for more time for the discussion was noted. Participants also noted the need for more balanced representation from adolescents as well as other family planning stakeholders, such as community members, especially in comparison to healthcare providers. Some participants were not comfortable with the language used. Young people felt older participants used complicated terminologies while community members felt the health care providers outnumbered them, in terms of representation.

**Conclusion:**

Generally, the community dialogue was well received by the community members and the healthcare providers, as was observed from the sentiments expressed by both categories. Some key considerations for refining the approach included soliciting maximum participation from otherwise marginalized groups like the youth would provide stronger representation.

## Background

At the London Summit for Family Planning in 2012, participation was appreciated as a key principle in ensuring that the goal of providing family planning access to 120 million additional women and girls by 2020, is met [1]. Community members’ views play an important role in shaping reproductive health practices [2]. As such, community-based participatory approaches leverage an understanding of the strengths and weaknesses among all the parties involved, as each party aims to solve a common problem, by combining knowledge and action for the social change needed to improve health and eliminate health disparities [3]. Research has shown the growing concern of the gap between evidence produced to improve health in communities [4], and the actual translation of this evidence into interventions and policies, especially among disempowered members of the communities [3]. Community participation, therefore, becomes a major contributor to knowledge generation and translation [3]. Participation is defined as the active involvement of affected populations in decision-making, implementation, management and evaluation of policies, programs and services [5].

Community dialogues are used as a participatory tool in the identification of rural health needs or to improve communication that encourages a common mutual understanding. Dialogues encourage the realization of perspectives and conditions of the other person or party, to identify and prioritize the needs of the community [6–9]. The dialogues aim to bring together parties that would not normally collaborate and assist them in aligning their thoughts on a particular issue [10]. A community dialogue forum allows the existing knowledge-base of each party to increase with consideration of the perspectives and views of others, and issues can be discussed and debated on in a neutral environment that allows a fair exchange of ideas [11].

Community dialogues have most commonly been used in HIV and AIDS prevention and mental healthcare programs [12–15]. In West Africa and other locations [8, 16], community group interventions have been used to shape healthy sexual and reproductive health behaviours [17]. In assessing whether a community participation programme could increase the met needs for family planning and contraception in Zambian communities [18], the UPTAKE Project tested the feasibility of a community dialogue involving health care providers and community members [18]. In the context of the project, the methodology (intervention) through which healthcare providers and the community were to be engaged was referred to as “The Approach” [9, 18–20].

The Approach was developed through well-informed evidence from existing literature [20] and refined through the integration of community, healthcare provider, and other stakeholders’ perspectives using qualitative research activities: focus group discussions (FGDs) and in-depth interviews during the formative phase of the Project [19, 21]. It defined best practices that addressed four stages in the implementation of a participatory programme: (i) programme initiation, (ii) participant identification and recruitment, (iii) ensuring community and health system dialogue and collaboration [9], and (iv) ensuring sustainability and scale-up [18]. The feasibility testing of the approach focused on stage (ii) and (iii), which involved participant identification and recruitment and ensuring community and health system dialogue and collaboration.

The Approach developed a rationale and hypothesized pathways that aimed to reduce the unmet need for contraception within a human rights framework (project Theory of Change, ToC) [9, 18, 22, 23]. The community dialogue methodology was structured around the project ToC, in a more simplified version, to encourage community and health system dialogue and collaboration in identifying possible causal pathways, as well as the requirements and assumptions that needed to be filled to achieve change. In other words, a simplified ToC framework served as a participatory tool to engage both community and health care providers to discuss and define possible causal pathways toward an overall outcome or desired change, which was to address the unmet need for family planning and contraception. They would then collaborate to implement, monitor and evaluate activities for each causal pathway.

The community dialogue approach was designed to assess the acceptability and feasibility of bringing together healthcare providers and the community to participate in discussions on improving Quality of Care, QoC in family planning and contraception provision. Early in the formative phase of the UPTAKE Project, QoC was identified as a key intermediate outcome that could lead to increased met needs for family planning and contraception [9, 18, 20, 24]. This was based on the assumption that quality services that respond to the needs of the users and potential users were a requirement for increased demand and utilization of services [25]. Further, considerations were integrated into the Approach following the *feasibility testing* and finalization of the formative phase research [18]. This paper reports on the feasibility testing of the community dialogue, which aimed to facilitate dialogue between the health care providers and community members, to increase the uptake of family planning and contraceptive services in Zambia.

## Methods

Establishing the feasibility of the community dialogue was undertaken in two ways: by conducting the community dialogue and evaluating it. Key stages of the dialogue (Additional file 2: Annexe 2), with representations from the community and health providers, were undertaken. An evaluation of the community dialogue through observations using a standardized feasibility testing tick-list (Additional file 1: Annexe 1) for all observers took place during the dialogue. Also, the feasibility testing evaluation included focus group discussions, FGDs with three groups of stakeholders who had participated in the community dialogue (health care providers only, community members only and a mixed group of health care providers and community members).

The observations and discussions evaluated the operational and cultural feasibility of the dialogue, with focus on the following areas: perceived acceptability, demand or estimated use of the Approach, implementation or the extent or likelihood and manner in which it could be implemented as planned or proposed, practicality for deliverability based on resources, time, commitment, etc., adaptation or whether it was necessary to change contents of the proposed approach and finally, integration or the level of system change needed to integrate the proposal into existing settings [9].

### Recruitment of participants

The invitees for the community dialogue were a combination of people from a minimum of three main categories; community members, health care providers, and other stakeholders in family planning and contraception. All three were considered influencers of family planning and contraception decision-making, community participation and representation (Table 1). The recruitment of participants was facilitated by a local district medical officer, who engaged the district nursing officer. The nursing officer facilitated the recruitment of health system representatives: nurses, clinical officers and other family planning and contraception practitioners. The officer also recruited the community (volunteer) health systems representative groups such as the Safe Motherhood Action Groups (SMAGs), the Neighborhood Health Committees (NHCs), and the family planning and contraception users and the adolescents among other community members.

**Table 1:** List of invitees for the community dialogue

	Participants categories	Invited	Confirmed	Attended
Community members	Community Members (Including Community Health Committees and users)	6	5	5
	Adolescents	3	2	2
	Community Leaders	3	0	0
Healthcare providers	Health Care Providers- Managerial	5	4	4
	Health Care Providers- Frontline	7	6	6
Other stakeholders	Teachers	2	2	2
	NGOs	4	3	2
	**Total**	**30**	**22**	**21**

Apart from the health care providers and community members, other stakeholders in family planning and contraception were invited by the UPTAKE Project research team. These included the Non-Governmental Organizations (NGOs), the education sector representatives through the District Education Board Secretary (DEBS) and the District Commissioner (DC) - who unfortunately was unable to attend the meeting. In total, of the 30 people invited: 21 managed to attend the dialogue, meeting the 70% minimum requirement that was agreed upon by the project team. Of the 21 attendees, seven were male and 14 were female.

### Evaluation of the community dialogue approach- through observations and FGDs

#### Observations

Non-participant observations were used as a data collection method for the evaluation [26], where people who were not participants of the community dialogue were selected to observe the entire session. The observations provided a broad overview of the setting, as well as the entire community dialogue process. The observers carried out guided observations while the community dialogue was taking place using a standardized feasibility testing tick-list (see Additional file 1: Annexe 1). The observers included a project team member, a health system representative and a community member to ensure that diverse views were captured. The health system and community representatives were taking part in the project for the first time while the project team member was more aware of the objectives of the community dialogue activity. This was done to control the observer effect [26]. The three different types of observers controlled any selectivity that could occur if only one (type) of an observer was considered [26]. Observations were also triangulated with the other data collection methods, such as the FGDs.

#### Focus-group discussions

Following the community dialogue session, three FGDs were conducted to hear from the participants about their views on the entire community dialogue implementation process. The facilitators used a pre-defined guide to structure the discussion. The FGDs explored the following themes: representation in the community dialogue, orientation and ground rules, participation in the community dialogue, the dialogue on family planning and contraception and QoC and the technical, schedule, and cultural feasibility of the community dialogue approach. The FGDs, as a form of participatory evaluation, allowed the participants to air their views on each of the themes.

The community dialogue participants were split into three groups for the FGDs: the community member group, the health care provider group, and a mixed group with both the health care providers and the community members. The groups were split to ensure ease of discussion among the group members and they were given the freedom to be in any group provided it was the right category. Each of the focus groups had 5–8 participants. Not all the participants of the dialogue were part of the discussions as they opted not to, or had to leave and attend to their work.

#### Data analysis

FGDs were audio-recorded with participants’ permission, transcribed verbatim, and translated where interviews were done in the local languages. NVivo (Version 10, QSR International), was used to organize and manage the data while thematic analysis [27] was used to analyse the data. The data was read and re-read for familiarity. A code-list, mainly consisting of pre-determined, broad themes was developed based on the key areas in the feasibility testing tick-list (Additional file 1: Annexe 1). New emerging structural and analytical themes were iteratively added to the initial code-list, as data analysis progressed. The data were coded separately by two coders but routinely discussed, to address any coding biases and to enhance inter-coder reliability [19]. Participant observations were noted during the dialogue by project team members and this enabled the triangulation of findings from the FGDs. The three observers’ findings from the community dialogue tick-list (Additional file 1: Annexe 1) were also read, aggregated and triangulated with the other community dialogue findings.

#### Ethical considerations

The process of informed consent was fully adhered to. The informed consent components were discussed with the group, and individual written consent was obtained from each of the participants by members of the UPTAKE Zambia team, the Principal Investigator, Research Assistant, Data Manager or the other Facilitators. All participants were over 18 years. The entire study, including the feasibility testing, was reviewed by the WHO technical and ethics review boards (A65896). Local ethical clearance was obtained from the University of Zambia Biomedical Research Ethics Committee (UNZABREC) and permissions to work within the Zambian health system was sought from the Ministry of Health.

### Key findings

This section highlights the community dialogue implementation process. Thereafter, results according to the participant observations and from the FGDs are presented under the following headings: recruitment and identification (attendance), orientation and ground rules, participation in the community dialogue, the dialogue of FP/C, and QoC, technical, schedule and cultural feasibility.

#### The community dialogue process

The community dialogue was conducted at a location in a suburban district of the Lusaka, outside the project intervention district. The entire dialogue took 125 min, with a 20-min break. The dialogue sessions were divided among three facilitators who were project staff members, according to 3 sections: 1) Orientation, 2) Establishing ground rules, 3) refining the ToC approach (Additional file 2: Annexe 2).

#### Agendas for community dialogue meeting

The first presenter gave a detailed background to the project, its goals, and the aims of the community dialogue. This activity lasted 20 min. The participants were then asked to suggest some ground rules that they felt would guide community dialogue. The ground rules session took 15 min. The ground rules for the dialogue included: having respect for other people’s opinions, speaking loudly, setting mobile phones to vibrate or be silent, raising hands if they wanted a chance to speak, not having any side meetings, participating actively, staying on topic during the discussions, acknowledging that all the participants in the room were equal, confidentiality- no one’s opinion would be revealed after the meeting ended or any time thereafter. Reference was made to the ground rules throughout the dialogue. This was then followed by an FGD with the community dialogue participants to get their views on the entire dialogue process, and how it could be improved.

After setting the ground rules, the first facilitator presented the simplified ToC to the participants. This part aimed to show the participants how the discussions would lead to the overall outcome, which was to reduce the unmet need for family planning. To explain further how the ToC was going to be used, a simple dry-run was done. A discussion, led by the second facilitator, was introduced to identify the assumptions and causal pathways to improved QoC in family planning and contraception provision. The intermediate outcomes were then discussed, with activities and assumptions for each outcome. Some of the intermediate outcomes included: having qualified health workers, the availability of preferred methods at the health facilities, having more respect for clients and having more equipped family planning areas. This activity took 60 min.

Once the causal pathways, activities and assumptions had been identified, the third facilitator led a discussion on identifying the most feasible and acceptable pathways with regards to acceptability, demand, implementation, practicality, adaptation and integration of the pathways. This criterion was based entirely on each participant’s opinion and participants were encouraged to speak freely on the ‘best’ pathways that could achieve more than one intermediate outcome. This activity took 90 min. After the discussions using the ToC participatory-tool had been concluded, the first facilitator wound down the discussion with a summary of the community dialogue activity, concluded and thanked the participants for their active contributions throughout the discussions.

### Observations and focus-group discussion results

#### Recruitment and identification of dialogue participants

During the FGDs, while some health care providers felt that there was adequate representation in the community dialogue, the community members mentioned that the dialogues required more representative participants to produce even more relevant results. For example, there was a need for political leaders to also be represented. An adolescent said:

*“We were not represented properly because were outnumbered, others were more [than us], but we were just a few of us (…)”*

*[Community focus group discussion, Adolescent]*

The community members also mentioned a need for representation from the churches and other faith-based organizations since it was recognized that they played an important role in shaping reproductive health behaviours. The community members also stated that the Community Health Workers were well represented, but that the pharmacists were needed to bring out more information on the vastly experienced stock-outs of family planning and contraception supplies.

*“(…) I think to be specific, we should have some representatives from churches, yes, and from the political wing, the stakeholders they should have called them because they are part of it (the discussion on family planning and contraception) as well.”*

*[Healthcare provider focus group discussion, Healthcare Provider]*

From the observations, it was noted that the adolescents’ turn-out was less than the intended number (2 out of 3). A bigger proportion of community members did not attend., while almost all the health care providers invited attended the meeting.

#### Orientation and ground rules

The participants felt that ground rules were appropriate since they all took part in creating them. Participants reported that they were able to remind themselves and each other to follow the rules because they were displayed throughout the discussion. Participants appreciated the importance of the rules in maintaining organization and order throughout the dialogue. A participant had this to say:

*They (ground rules) came from the participants, it was not imposed on the participants, but it’s the participants that came up with those rules.”*

*[Mixed focus group discussion, Healthcare Provider].*

One of the young people, among the community members, on the other hand, felt that the rules should have been explained in more detail for him to understand what they meant. It was assumed that all the rules were understood because they came from the participants. An adolescent said:

*“I did not understand the first one, the one that says speak through the chair, and do you speak alone or…?”*

*[Community focus group discussion, Adolescent].*

#### Participation in the community dialogue

During the dialogue, there was a consensus among the participants that English was suitable for the articulation of ideas by participants, whether they were from the health sector or not. However, some community members during the FGDs mentioned they could have expressed themselves better in the local language despite agreeing to use English during the dialogue session. Other members of the community felt as if the discussion was specialized or technical, especially when the health care providers spoke. The use of technical language and abbreviations during the dialogue was discussed during the FGDs. With regards to the presentations during the dialogue, community members indicated that where some acronyms they did not understand, hence on some occasions, they could not answer appropriately.

*”.. the language; it is not everyone that has been to school or up to grade12, at least there was supposed to be a mix up of language with English because some of us work in the community and we use local languages. So somehow to some of us, some words were big (complicated or unfamiliar jargon).”*

*[Community focus group discussion, Community Member].*

During the FGDs, young people also mentioned that they felt English was the most appropriate language to use. However, some terminologies and acronyms were complicated for them but they felt too shy to ask.

*“Like the term ‘integration’, that term yes was not clear.”*

*[Community focus group discussion, Adolescent].*

A community member, who sat next to a young person during the dialogue also felt that the youth were unable to ask what the terms meant in the larger group. It was much easier for them to inquire from their neighbours in the meeting and not through the moderator.

*“Oh yeah, okay they (terminologies) were coming from us participants and some community health workers because those are normal terminologies, but it might have been different from teachers, those are not like, this man (name mentioned) am sure he is very aware of them, but there are community workers who are from teaching maybe like my son here, the adolescent, he is not familiar with those terminologies.”*

*[Healthcare provider focus group discussion, Healthcare provider].*

From the observations and interviews, community members participated well in the community dialogues despite being outnumbered by the health care providers in the meeting. Overall, both the community members and the health care providers, as well as other stakeholders, reported during the FGDs that they felt free to speak their minds and participate as this was reinforced by one of their ground rules (having to no wrong answers). One health care provider had this to say:

*“I feel there were no barriers because people were able to express whatever they wanted to say and no one was opposing even when the answer that one gives or opinion that one gives was not opposed …*”.

*[Mixed focus group discussion, Healthcare provider].*

A community member also said:

*“Actually, it (the dialogue) was something that was good, it is important once in a while to meet together with the community and the health providers because, for health providers, there is nothing that we can do without the community, cause or anything sensitization and other things we depend on the community so the community should be given information on everything that is there in the health facility. So we are represented, it’s a link actually between the community and the health Centre, and so these representatives are important to us and the health providers because they are the ones who speak to us and whatever we want from the community we ask these people to speak for us.”*

*[Community focus group discussion, Community Members].*

It was observed that the adolescents also expressed themselves by bringing out issues that affected their access to family planning and contraception information and services. However, they were not entirely free to speak and required active encouragement from the facilitators. This was attributed to their discomfort speaking out in a public forum and feeling outnumbered by the adults.

### Dialogue on family planning and contraception and QoC

Despite the feeling that the health care providers were more familiar with the topics being discussed, the community members felt able to express their views on the subject of QoC in family planning and contraception. A healthcare provider said:

*“I think it was okay, we have interacted freely, openly and we have understood each other’s views, and from this, I think we are picking a leaf for forward (to move forward) in life”.*

*[Healthcare provider focus group discussion, Healthcare providers].*

FGD participants agreed that access to family planning and contraception information and services was vital. They agreed that this was an area that needed to be deliberated so that overall unmet need could be reduced. The participants also mentioned the benefits of family planning and contraception and that there was more work to do regarding sensitizing the community about the different options they had. They made specific reference to the need for more ‘appropriate’ information and services among the youth.

*“I would concur with her (fellow participants) because of the unwanted pregnancies mostly, and they end up to have unsafe abortions, so if the services are given to these adolescents, I think it will prevent the abortions and unwanted pregnancies among the youth.”*

*[Healthcare provider focus group discussion, Healthcare provider].*

Conversely, an education sector representative expressed reservations, especially regarding the dissemination of all family planning and contraception information with the youth. The representative felt adolescents were too young and that this information needed to be altered to make it more age-appropriate. Without such censoring, the information was seen to increase the rates of promiscuity among the youth.

Despite such dissent, all the participants agreed that the dialogue allowed them to learn from each other, and they agreed that they had different viewpoints concerning QoC- health care providers viewed quality from the supply side while community members viewed it from the demand side. For example, the lack of certain services such as the Intra-Uterine Device was attributed to staff shortages and not negligence on the health care providers’ part. This was discussed during the dialogue.

*“I would say it was good because there were some divergent views before we could come to agree because we were also given opportunity to argue, and try to raise some concerns, challenges that we need, and also what the community is complaining, but at last we agreed to say we have to work together, we need to prioritize our systems and working so that both of us benefit.”*

*[Community focus group discussion, Community Member].*

From the observations, some strong differences in opinion about QoC were noted between the community members and the health care providers. During the FGD discussion, participants concluded that they were looking at QoC, but from different perspectives—the supply side and the demand side.

*“(...) yes I think this was a very good discussion, this one has helped since we have people from the district office: education, it will help them plan, how can we get out there to the children, how can we reach them with this information and we also heard their blocks/obstacles and all this. So we didn’t know as health providers that we also have a limit. We thought it was easy for us to go and talk about sexuality openly in their schools. So I think it was a very good thing that at least even as health workers will know how we are going to take it when we meet a child who is ours and will not behave like a parent but as a health service provider(...)”*

*[Mixed focus group, Healthcare provider].*

When asked about their view on the importance of the discussion on QoC, all the participants reported that they agreed on both the definition and the key constitutes of QoC. They could explain what they thought should constitute QoC even though a single definition was still a challenge to achieve among the various stakeholders invited. A young person felt he expressed his fellow young peoples’ views- that the healthcare provider needed to be more welcoming at health care centres.

*“It was useful such that it kind opened up on how the people in the health centres talk to the adolescent when they come to ask for help from them like if you want to ask for a condom, they shouldn’t go like*” *as young as you are” and all.”*

*[Community focus group discussion, Adolescent].*

A health care provider said;

*“I think we did agree on what Quality of Care is as a community and the health care providers and that’s the more reason why we came up with those terms, yes we were very free to express ourselves and even after expressing ourselves although seemingly we were putting the health provider in a squeeze position but no it was so nice that at least both parties were able to understand, where each one of us is coming from and then at the end of the day we fully realized how important and the stressful conditions that this other person is in.”*

*[Mixed focus group discussion, Healthcare providers].*

#### Schedule feasibility

It was observed that most of the participants found it difficult to get acquainted with the topics being discussed. As the participants got more familiar with the dialogue topics, and what was needed of them, the meeting became more interactive. Some community members in the FGDs noted the need for more time to be allocated to the discussions. A need for more time was requested, to provide the participants with more room to express all their opinions and ideas. It was reported that since participants were from different locations, each one of them needed to be actively given a chance to take part in the dialogue. The time limitation was attributed to the pace of the richness of the discussion. Participants felt that the activity was much clearer towards the end of the allocated time for the discussion. This is when they felt even more encouraged to express their opinions.

*“The duration of the meeting was … we needed to be here for two days or so.”*

*[Community focus group discussion, Community Member].*

On the other hand, a few community members felt the time allocated to the dialogue was sufficient. However, certain participants (healthcare providers) had too much time to speak compared to the others and that there needed to have been some control in the time allowed to speak.

“*Yes, I think it was ok except that other people dominated the floor, and I feel other people would have also benefited to make their views known but some people took most of the time.”*

*[Community focus group discussion, Community Member].*

Interestingly, the health breaks between sessions were appreciated, as it was noted that they allowed participants to refresh themselves.

#### Cultural feasibility

Though mentioned briefly, all the participants felt the community dialogue approach was culturally sound. This is because it allowed them to address a common problem; reducing problems associated with unmet need for family planning and contraception. A community member further added that there was a need for more representation from the community to further determine the cultural acceptability of the Approach. In other words, if more representatives from different sectors were invited to share their views, then the cultural acceptability would be clearer. They said:

*“The approach was quite okay like we are saying, we know society is dynamic and we move with time and trends that we have previewed at that particular time, but am sure like we heard from the young man, he said he could not come so easily because he was amongst the elders, because our tradition says that they are certain things that we can easily mention in the public, others we cannot. Although in this forum, it wasn’t coming out like hinting so much on our tradition.*

*[Community focus group discussion, Community Member].*

However, others still felt that the approach dealt with an issue that was seen to potentially be problematic to discuss if certain members of the dialogue were present or had more representation.

*“I feel it [the approach] cannot be applied especially in the religious context, for instance, if the girls are introduced to using family planning, it is like you are telling them to start engaging in sex. Whereas religious in the bible the bible says children not to start engaging [in sex] until they get married. So if you give them contraceptives, it is like you are telling them to go ahead and have sex.” [Community and Healthcare Provider HCP].*

#### Technical feasibility: the use of visual aids during the community dialogue

In the FGDs, participants mentioned the usefulness of the aids used during the dialogue: the flip chart, the one-pager documents with information of QoC and the unmet need for family planning and contraception, and the PowerPoint presentations, especially during the discussion on the different pathways, activities and assumptions.

*(...) because we were able to get everything they were teaching there (able to use the visual aids).*

*[Community Member focus group discussion, community member].*

The participants also felt that the aids used were able to open up their minds to the broader project context, and give them even better ideas about what would or would not work in the Zambian context. Community members and health care providers were able to understand more about what the aim of the dialogue session was.

*“I am talking in terms of how the program started because we were using PowerPoint we were able to see the definition, we would start by introducing the background of the project itself, we learnt that the project is running in three countries so we were able to understand (...).”*

*[Healthcare provider focus group discussion, Healthcare providers].*

## Discussion

This paper aimed to document the feasibility of a community dialogue approach bringing together health care providers and the community to reduce the unmet needs for family planning and contraception in Zambia. Factors that influenced the implementation of the community dialogue during the community dialogue and subsequent evaluation through FGDs are highlighted in the discussion.

### Positive attributes of the community dialogue

The participants generally agreed that family planning and contraception were important issues that needed to be discussed, despite coming from different backgrounds. They understood that key barriers to the provision of family planning and contraception services, as well as uptake in the community needed to be explored and addressed. Such success has been reported in other dialogues in family planning, suicide and HIV prevention as they aim to achieve a common understanding of the problem [12, 15, 28]. The dialogue ensured a shared understanding of the problem and the steps that needed to be taken for that problem to be solved.

Participants mentioned that they learned more than they knew about family planning and contraception before the dialogue, a finding similar to Tesfaye in Ethiopia on curbing HIV using community dialogues who found that this was a key driver of implementation [13]. Adding to the evidence in Zambia on participatory interventions in family planning and contraception, this study suggests the feasibility of the Approach and the possibility of the success of such a complex-designed intervention in the Zambian context [2, 20].

Divergence in the categories of people invited enriched the meeting as it facilitated increased representation from the community and from the health sector too, similar to what was found in South Africa [9]. The dialogue approach facilitated the engagement of the community and health care providers as it was viewed as feasible, despite cultural influences that shape family planning and contraception outcomes. The use of a community dialogue forum or platform for the different stakeholders allowed a deeper understanding of the other participants’ views, who they previously blamed for hurdles in service provision. However, a need to be proactively aware of the power dynamics [20]- especially where the healthcare providers were more conversant with the subjects under discussion was noted. A more inclusive approach to sharing time on the floor to air opinions is suggested.

Regarding orientation and ground rules, the process was appreciated as this triggered and ensured ownership of the rules by the participants. Since the participants took part in the development of the rules, share in feelings of the rules being imposed on them by outsiders (facilitators). The rules were thus acceptable and were adhered to.

### Considerations for refining the approach

Adequate representation ensured that divergent and varied viewpoints were considered and in the discussions, and for a new shared understanding to be developed. The importance of representation from a wide range of stakeholders was stressed in other community dialogues however, this could only be addressed after an evaluation of the dialogue, and suggestions for more stakeholders were made [9, 12, 13].

A consideration for refining the approach was the engagement of the religious and community leaders in the dialogue. Also, considering that all the health system participants attended the meeting compared to the community members, inviting more community members could actively increase representation. Besides, the adolescents also felt that they were outnumbered by the adults. Another consideration for the success of other such dialogues involving the youth would be to conduct separate dialogues for the young people, but still have them represented in the larger dialogue sessions. This would be a key response to the unbalanced power relations between the younger and older participants.

A need to gain more insights from the actual users of family planning and contraception services was mentioned. In this case, the participants were selected and invited into the discussion as ‘representatives’ from the community but not really as ‘users’. Therefore, the user perspectives were missing in the discussion. During the preparations, efforts were made to identify various categories of stakeholders, and more potential participants were suggested during the dialogue, amplifying the importance of the feasibility testing itself.

Allowing more time for the discussions was suggested as participants were more eager to express their views as they got more familiar with the topic. For more meaningful dialogue and understanding of the shared problem, there was a need for more time to discuss the causal pathways to change. This is similar to Tesfaye’s finding in Ethiopia [13], though this study had over 60 participants, which is over twice the number of participants invited to the feasibility testing. This points out the need to be balanced in the total number of participants invited to the dialogue and to be mindful of the time taken for the overall dialogue [9].

The language used required more consideration, as some participants may not have been as eloquent as was thought with one language. The consideration of using clear and direct language was made during the discussion on ground rules. Much as the team thought the language was addressed, there was still a need to address terminologies and to continuously assess the ease of use of one language during the discussion. Crankshaw et al. (2019) in South Africa found that bilingual notetakers during the dialogue process were useful in facilitating language complexities [9]. Also, the use of multilingual facilitators could have addressed the language barriers encountered. A study to facilitate dialogue in HIV revealed that language was considered before the meeting, and all the facilitators were multilingual, according to the community targeted [13]. Active usage of more than one language proved useful.

The facilitators of the community dialogue meeting needed more training on using the ToC tool to facilitate the meeting. This would reinforce the importance of creating the space for dialogue, ensuring the respectful expression of diverging views, and to fully understand the desired outputs of the intervention.

### Limitations and strengths

One of the limitations of this feasibility testing is that it was a one-off meeting, and some of the other components of the Approach were not tested during this meeting. Also, some of the issues, such as the cultural feasibility of the approach, could have been explored further in subsequent meetings. However, the feasibility testing (community dialogue) stage of the intervention was a vital stage in the development of the Approach and valuable information was obtained from the evaluation.

Regarding the methodology, some observations may have brought out some subjective findings [26]. This was addressed by selecting three different types of observers [29] to clarify any observer biases. These findings were also triangulated with the findings from the FGDs [29]. Finally, while the feasibility testing was nested in a participatory approach that aimed to bring together healthcare providers and the community members, this refinement stage of the development of the intervention achieved the “the edge effect,” as coined by Burton and Keagan (1999) [30]. This means the community benefited from the knowledge and contact facilitated by the research project by meeting, interacting and sharing information for mutual growth and benefit to all members of the community.

## Conclusion

The participants of the feasibility testing accepted the community dialogue approach. The participants were able to deliberate solutions to a shared problem and they had enriched views due to the diversity of representation during the dialogue. Despite coming from different backgrounds and having different viewpoints, this platform allowed them to agree on the importance of and have a shared understanding of the problem-high unmet need for family planning and contraception. Participants suggested solutions that were acceptable to all the participants despite their differences, and dissenting views were also allowed and considered. It is important to note, however, that certain factors need to be taken into consideration to yield more rich discussions. Great emphasis on language of choice is stressed and discussions would benefit from multilingual facilitators. A need to identify and prioritize views from more marginalized persons such as the adolescents, in this case, would prove essential too.

## Supplementary information

**Additional file 1: Annexe 1.** Feasibility Testing Tick-list.

**Additional file 2: Annexe 2.** Feasibility Testing Agenda.

## Data Availability

The data are not publicly available as it contains information that could compromise research participant privacy/consent. However, some anonymized aspects of the datasets may be available upon request and with permission of the Department of Sexual and Reproductive Health and Research, World Health Organization. Note that data sharing is subject to WHO data sharing policies and data use agreements with the participating research centers.

## Abbreviations

DC: District Commissioner
DEBS: District Education Board Secretary
FGDs: Focus Group Discussions
NGOs: Non-Governmental Organizations
QoC: Quality of Care
SMAGS: Safe Motherhood Action Groups
ToC: Theory of Change
WHO: World Health Organization
