# Supplementary material for: Feasibility testing of a community dialogue approach for promoting the uptake of family planning and contraceptive services in Zambia
Source: BMC Health Serv Res. 2020 Aug 8;20:728. doi: 10.1186/s12913-020-05589-5 (PMC7414985; doi:10.1186/s12913-020-05589-5)
Supplement: Supplementary file 1 — Additional file 1: Annexe 1. Feasibility Testing Tick-list. [file 12913_2020_5589_MOESM1_ESM.docx]

Additional file 1

*Annexe 1: Feasibility Testing Tick-list (COMMUNITY DIALOGUE)*

*This tick-list will be filled in by at least three members of the Project team, who are the facilitator, note-taker and other members (evaluator), present during the community dialogue during or directly following the community dialogue before the Focus Group Discussion. The aim is to establish the operational feasibility of the Approach, as well as the technical, schedule and cultural feasibility. This will also provide some input on what questions to focus on and explore deeper during the Focus Group Discussions and their analysis.*

| **Number** | **Question** | **Response** |
| --- | --- | --- |
| \| GENERAL INFORMATION \| \| \| \| \| \| \| --- \| --- \| --- \| --- \| --- \| --- \| \| 001 \| Name : \| _______________________________ \| \| \| \| \| 002 \| Role: \| _______________________________ \| \| \| \| \| 003 \| Date conducted: \| \|  \|  \| / \|  \|  \| / \|  \|  \| *dd/mm/yy* \| \| --- \| --- \| --- \| --- \| --- \| --- \| --- \| --- \| --- \| \| \| \| \| \| 004 \| Start and End time of the Community Dialogue \| \|  \|  \| : \|  \|  \| - \|  \|  \| : \|  \|  \| \| --- \| --- \| --- \| --- \| --- \| --- \| --- \| --- \| --- \| --- \| --- \| \| \| \| \| \| 005 \| Number of minutes per activity:  Orientation  Making Ground Rules  Community and Healthcare provider dialogue \| \|  \|  \|  \| minutes \| \| --- \| --- \| --- \| --- \| \|  \|  \|  \| minutes \| \|  \|  \|  \| minutes \| \| \| \| \| \| *OPERATIONAL FEASIBILITY*  RECRUITMENT \| \| \| \| \| \| \| 006 \| Number of community dialogue participants: \| \|  \|  \| \| --- \| --- \| \| \| \| \| \| 009 \| Were community members represented? \| YES………………………………  NO………………………………… \| □  □ Review recruitment  process \| \| \| \| 010 \| Number of community participants: \| all beneficiaries (users and non-users; women and men)  marginalized (adolescents or representative or migrant groups)  community representatives \| \| \| \|  \|  \| \| --- \| --- \|  \|  \|  \| \| --- \| --- \|  \|  \|  \| \| --- \| --- \| \| \| 011 \| Was the health system represented? \| YES………………………………  NO………………………………… \| □  □ Review recruitment  process \| \| \| \| 012 \| Number of healthcare provider: \| hospital/regional/clinic manager or representative  direct health care provider \| \| \| \|  \|  \| \| --- \| --- \|  \|  \|  \| \| --- \| --- \| \| \| 013 \| Were other stakeholders represented? \| YES…………………………………  NO………………………………… \| \| □  □ Review recruitment  process \| \| \| 014 \| Number of stakeholders: \| reproductive health NGOs, international organizations  other organizations (advocacy group, civil society or teachers) \| \| \| \|  \|  \| \| --- \| --- \|  \|  \|  \| \| --- \| --- \| \| \| 015 \| Was informed consent obtained from the participants? Signed informed consent is required for the FGD. For community dialogue, informed consent means, that during recruitment process potential participants were informed: of the aim of the activity what will happen during the activity of the possible risks that they can refuse to participate \| YES………………………………  NO……………………………… \| □  □ Stop study \| \| \| \| 016 \| Were minority or marginalized groups represented?  Specify: \| YES…………………  NO…………………… \| □  □ Explore reasons in FGD \| \| \| \| 017 \| Was there adolescent representation?  Specify: \| YES………………………………  NO…………………………………  _____________________  _____________________ \| □  □ Explore reasons in FGD \| \| \| \| ORIENTATION AND GROUND RULES \| \| \| \| \| \| \| 018 \| Did facilitators explain the aims of the Community Dialogue? \| YES………………………………  NO………………………………… \| □  □ Explore reasons in FGD \| \| \| \| 019 \| Did participants understand the aims of the Community Dialogue? \| YES………………………………  NO………………………………… \| □  □ Explore reasons in FGD \| \| \| \| 020 \| Did the community dialogue participants agree on ground-rules? \| YES………………………………  NO………………………………… \| □  □ Review approach \| \| \| \| COMMUNITY DIALOGUE BETWEEN COMMUNITY AND HEALTHCARE PROVIDERS \| \| \| \| \| \| \| 021 \| Did the community members participate in the dialogue? \| YES………………………………  NO………………………………… \| □  □ Explore reasons in FGD \| \| \| \| 022 \| Did healthcare providers participate in the dialogue? \| YES………………………………  NO………………………………… \| □  □ Explore reasons in FGD \| \| \| \| 023 \| Did community members and healthcare providers discuss with each other? \| YES………………………………  NO………………………………… \| □  □ Explore reasons in FGD \| \| \| \| 024 \| Did facilitators use language that is appropriate to participants? \| YES………………………………  NO………………………………… \| □  □ Explore reasons in FGD \| \| \| \| 025 \| Did participants report being at ease to communicate their views? \| YES………………………………  NO………………………………… \| □  □ Explore reasons in FGD \| \| \| \| Dialogue on FP/C and Quality of Care \| \| \| \| \| \| \| 026 \| Was the topic of Quality of Care in FP/C discussed? \| YES………………………………  NO………………………………… \| □  □ Review approach \| \| \| \| 027 \| Were QoC definitions discussed? \| YES………………………………  NO………………………………… \| □  □ Explore reasons in FGD \| \| \| \| 028 \| Were the community members able to express their views on QoC? \| YES………………………………  NO………………………………… \| □  □ Explore reasons in FGD \| \| \| \| 029 \| Were HCPs willing to discuss QoC? \| YES………………………………  NO………………………………… \| □  □ Explore reasons in FGD \| \| \| \| 030 \| Was there a consensus on QoC definitions \| YES………………………………  NO………………………………… \| □  □ Explore reasons in FGD \| \| \| \| 031 \| Did the participants agree that FP/C is important? \| YES………………………………  NO………………………………… \| □  □ Review approach \| \| \| \| ***technical, schedule and cultural feasibility of the approach*** \| \| \| \| \| \| \| 032 \| Was the approach able to recruit participants within the timeframe (planned recruitment process was approximately 1.5 months)? \| YES………………………………  NO………………………………… \| □  □ Explore reasons in FGD \| \| \| \| 033 \| Did more than 70% of the confirmed participants attend? \| YES………………………………  NO………………………………… \| □  □ Explore reasons in FGD \| \| \| \| 034 \| Was the duration of the meeting perceived as appropriate? \| YES………………………………  NO………………………………… \| □  □ Explore reasons in FGD \| \| \| \| 035 \| Was the budget sufficient to implement the meeting? \| YES………………………………  NO………………………………… \| □  □ Explore reasons in FGD \| \| \| \| 036 \| Was the approach perceived to be appropriate in the cultural context? \| YES………………………………  NO………………………………… \| □  □ Explore reasons in FGD \| \| \| \| 037 \| Were the materials (resources or tools) used understandable? \| YES………………………………  NO………………………………… \| □  □ Explore reasons in FGD \| \| \| \| 038 \| Did the materials (resources or tools) used initiate desired dialogue? \| YES………………………………  NO………………………………… \| □  □ Explore reasons in FGD \| \| \| | | |
