# Supplementary material for: Feasibility testing of a community dialogue approach for promoting the uptake of family planning and contraceptive services in Zambia
Source: BMC Health Serv Res. 2020 Aug 8;20:728. doi: 10.1186/s12913-020-05589-5 (PMC7414985; doi:10.1186/s12913-020-05589-5)
Supplement: Supplementary file 2 — Additional file 2: Annexe 2. Feasibility Testing Agenda. [file 12913_2020_5589_MOESM2_ESM.docx]

Additional file 2

*Annexe 2: Feasibility Testing Agenda (COMMUNITY DIALOGUE)*

**UPTAKE PROJECT**

**Community Dialogues Programme**

**17^th^ February 2016**

| **TIME** | **ACTIVITY / TOPIC** | **PERSON RESPONSIBLE** |
| --- | --- | --- |
| **Wednesday, 17^th^ February 2016** | | |
|  | **Community Dialogues** |  |
| 08:00 – 08:15 | Registration | Admin Assistant |
| **Chairperson for Morning Session** | | Co- Principal Investigator |
| 08:15 – 08:30 | WELCOME REMARKS | District Medical Officer |
| 08:30 – 09:00 | BACKGROUND TO UPTAKE PROJECT  Project Goals and Problems in Family Planning service delivery  Objectives of Community dialogues | Principle Investigator |
| 09:00 – 09: 30 | Presentation on Theory of Change | Principal Investigator |
| 09:30 – 10:15 | Plenary Discussion | Facilitator, UPTAKE Project |
| 10:15 – 10:30 | **HEALTH BREAK** |  |
| **Chairperson, Mid-morning Session** | | Facilitator, UPTAKE Project |
| 10:30 – 11:30 | Discussion on Quality of Care (QoC)  Identify assumptions and requirements for QoC  Identify intermediate outcomes, possible pathways & relevant activities | Facilitator, UPTAKE Project |
| 11:30 – 12:30 | Discussion to identify the most feasible and acceptable pathways to enhance QoC | Research Assistant |
| 12:30 – 13:00 | Summary from Community Dialogue | Principal Investigator |
| 13:00 – 14:00 | **LUNCH** | Admin Assistant |
| **Chairperson, Afternoon Session** | | Facilitator, UPTAKE Project |
| 14:00 – 15:00 | Focus Group Discussions (3 groups) | Facilitator, UPTAKE Project |
| 15:00 – 15:30 | Summary of Proceedings of the day | Facilitator, UPTAKE Project |
| 15:30 – 15:45 | CLOSING REMARKS | Principal Investigator |
| 15:45 – 16:00 | **HEALTH BREAK** | Admin Assistant |
| 16:00 | **END OF PROGRAMME** |  |
